# Virtual Reality Distraction during Endoscopic Urologic Surgery under Spinal Anesthesia: A Randomized Controlled Trial

**DOI:** 10.3390/jcm8010002

**Published:** 2018-12-20

**Authors:** Jee Youn Moon, Jungho Shin, Jaeyeon Chung, Sang-Hwan Ji, Soohan Ro, Won Ho Kim

**Affiliations:** 1 Department of Anesthesiology and Pain Medicine, Seoul National University Hospital, Seoul 03080, Korea; snu23802@snu.ac.kr (J.Y.M.); stjan24@gmail.com (J.S.); jychung1991@snu.ac.kr (J.C.); taepoongshin@gmail.com (S.-H.J.); nosuan@gmail.com (S.R.); 2 Department of Anesthesiology and Pain Medicine, Seoul National University College of Medicine, Seoul 03080, Korea

**Keywords:** virtual reality, sedation, spinal anesthesia, endoscopic urologic surgery

## Abstract

Sedation protocols during spinal anesthesia often involve sedative drugs associated with complications. We investigated whether virtual reality (VR) distraction could be applied during endoscopic urologic surgery under spinal anesthesia and yield better satisfaction than pharmacologic sedation. VR distraction without sedative was compared with pharmacologic sedation using repeat doses of midazolam 1–2 mg every 30 min during urologic surgery under spinal anesthesia. We compared the satisfaction of patients, surgeons, and anesthesiologists, as rated on a 5-point prespecified verbal rating scale. Two surgeons and two anesthesiologists rated the scale and an overall score was reported after discussion. Thirty-seven patients were randomized to a VR group (*n* = 18) or a sedation group (*n* = 19). The anesthesiologist’s satisfaction score was significantly higher in the VR group than in the sedation group (median (interquartile range) 5 (5–5) vs. 4 (4–5), *p* = 0.005). The likelihood of both patients and anesthesiologists being extremely satisfied was significantly higher in the VR group than in the sedation group. Agreement between the scores for surgeons and those for anesthesiologists was very good (kappa = 0.874 and 0.944, respectively). The incidence of apnea was significantly lower in the VR group than in the sedation group (*n* = 1, 5.6% vs. *n* = 7, 36.8%, *p* = 0.042). The present findings suggest that VR distraction is better than drug sedation with midazolam in terms of patient’s and anesthesiologist’s satisfaction and avoiding the respiratory side effects of midazolam during endoscopic urologic surgery under spinal anesthesia.

## 1. Introduction

Virtual reality (VR) technology has been investigated for its clinical applications [1,2]. Studies have investigated the educational use of VR for simulation-based training [3,4,5,6,7]. VR has also been evaluated as a non-pharmacological means of mitigating acute procedural pain and has also been demonstrated to be effective for the attenuation of pain perception, anxiety, and general discomfort in both adults and children [1,8,9,10,11]. 

VR may distract patients from noisy, scary, and uncomfortable environments during surgery under spinal or regional anesthesia. A recent pilot study investigated whether immersive VR therapy can be used as an adjunctive nonpharmacolgic sedating strategy in patients who receive drug sedation with propofol [12] and found no significant difference in the propofol requirement between the groups, although there was less propofol consumption in the VR group [12]. However, the effect of VR distraction alone under spinal anesthesia has not been evaluated. It is still unknown whether VR can provide safe and effective distraction and replace pharmacologic sedation.

During endoscopic urologic surgery under spinal anesthesia, the patient may receive pharmacologic sedation to lessen the effect of noise in the operation room, the unpleasantness of the procedure, and the fear of surgery. However, sedative drugs may cause complications, such as respiratory depression or hemodynamic instability [13,14]. Distraction by a VR program that includes visual and audio suggestions for relaxation may achieve the intended goals of pharmacologic sedation without drug-related side effects. 

Therefore, we hypothesized that VR distraction might achieve greater patient satisfaction without the side effects of drug sedation during urologic surgery under spinal anesthesia. To this end, we conducted a randomized controlled trial comparing patient satisfaction between VR distraction and midazolam-based sedation in patients undergoing endoscopic urologic surgery under spinal anesthesia.

## 2. Methods

### 2.1. Study Design

The protocol of this prospective, parallel, non-crossover, single-blind, randomized controlled clinical trial was approved by the Institutional Review Board of Seoul National University Hospital (No. 1611-069-808) and was registered prior to patient enrollment at http://www.clinicaltrials.gov (NCT03055663, Principal investigator: Won Ho Kim, data of registration: 14 February 2017). All patients provided written informed consent. This study was conducted at the Seoul National University Hospital, Seoul, Republic of Korea between March and November of 2017. 

### 2.2. Patients

The eligibility criteria were scheduled endoscopic urologic surgery, including holmium laser enucleation of the prostate (HOLLEP) or transurethral resection of bladder tumor), and an American Society of Anesthesiologists (ASA) physical status classification of I–III. The exclusion criteria were as follows: history of chronic use of sedatives or narcotics (>6 months), alcohol or drug abuse, baseline pulse oximetry saturation of less than 90%, and baseline hemodynamic or respiratory instability (initial systolic arterial pressure <80 mmHg, respiratory rate >25 or <10 breaths/min). Medication history was screened in all patients and chronic user of the listed drugs was excluded because the response to the midazolam in these patients may differ. Patients were randomly assigned to a VR group or a sedation group. Randomization was conducted by a number table generated from an internet-based computer program (www.randomization.com). A staff anesthesiologist who is independent of our study made a random allocation sequence, enrolled participants and assigned participants to interventions. The study participants were allocated using sequentially numbered, opaque, sealed envelopes containing random allocation numbers. The sealed envelope was opened after induction of anesthesia by the attending anesthesiologist, who was not one of the trial investigators. Surgeons who reported their satisfaction score and the outcome assessor who measured the satisfaction score of the patients in the recovery room were blinded to group assignment. The patients and attending anesthesiologists could not be blinded to group assignment.

### 2.3. Anesthesia Protocol 

All patients underwent routine monitoring, including electrocardiography, non-invasive arterial pressure monitoring, pulse oximetry, and capnography. Mean arterial pressure, respiratory rate, and heart rate were measured every 3 min and oxygen saturation (SpO_2_) and end-tidal CO_2_ concentration (ET_CO2_) were monitored continuously. No premedication was administered. Spinal anesthesia was induced by intrathecal injection of hyperbaric bupivacaine 12–14 mg with fentanyl 20 µg at the L4–L5 or L5–S1 spinal level to achieve a spinal sensory block level of at least T10. After induction of spinal anesthesia, all patients received their allocated study intervention. All patients were observed postoperatively in the recovery room for at least 30 min.

### 2.4. VR Group

The patients assigned to the VR group were subjected to a 30-min VR program (Aqua 30 Korean Version 4.0, Oncomfort SA, Wavre, Belgium) via a head-mounted display and earphone connected to an android cell phone (Galaxy 7.0, Samsung, Seoul, Korea) (Supplemental Figure S1). This program was designed for relaxation and distraction from anxiety or pain of patients (www.oncomfort.com/en). The patients watched a VR underwater view of the ocean while listening to narrations designed to induce relaxation and meditation. If the surgery was not completed within 30 min, the patient watched the VR program again. Details of the narration are provided in Supplemental Text S1 and screenshots are shown in Supplemental Figures S2–S4. The sedation group underwent pharmacological sedation with midazolam using an initial bolus dose of 1–2 mg and a maintenance dose of 1–2 mg every 10–30 min. Oxygen was supplied at 5 L/min via a facemask in the sedation group but not in the VR group. However, to blind the surgeon to group assignment, the VR headset and earphone were also used in the sedation group and a facemask was also used but without an oxygen supply in the VR group. If a patient assigned to the VR group wanted to stop watching the VR program, propofol could be administered as rescue medication and the incidence of rescue medication was recorded.

### 2.5. Study Outcomes 

Baseline demographic data, including age, sex, and body mass index, were recorded preoperatively. The type of surgery and the total operating time were also recorded. The primary outcome variable was the patient-reported satisfaction score measured by a 5-point Likert-like verbal rating scale according to a prespecified score-defining table (1, extremely dissatisfied; 2, dissatisfied; 3, undecided; 4, satisfied; 5, extremely satisfied; Table 1). Patient’s satisfaction score was measured at the recovery room when the patient became alert. The surgeon’s and anesthesiologist’s satisfaction scores were also measured at the end of surgery according to the same prespecified score table (Table 1) as secondary outcomes. The criteria for reporting the satisfaction score were explained to the patients, surgeons, and anesthesiologists before induction of anesthesia. To minimize reporting bias, two surgeons and two anesthesiologists were involved in reporting the satisfaction score. These two surgeons and two anesthesiologists discussed and reported a single satisfaction score. The two anesthesiolgists included the attending anesthesiologist and a supervising staff anesthesiologist, who were not otherwise involved in the study. Any discrepancy in the satisfaction score was resolved by discussion and a single overall satisfaction score was reported for each patient. The assigned surgeons and anesthesiologists did not change throughout the study period.

The incidence of patient discomfort intraoperatively for any reason, requests by patients to stop watching the VR program, and unnecessary patient movements were recorded. Episodes of SpO_2_ desaturation (<90% for more than 5 s), apnea (undetectable ET_CO2_ for more than 5 s), and administration of rescue sedation with propofol (if an optimal level of sedation was not achieved, as judged by agitation or intolerance) were also recorded. Hypotension was defined as a decrease in mean arterial pressure to <55 mmHg or up to a 20% reduction from baseline and bradycardia was defined as a heart rate <50 beats/min or a 20% reduction from baseline. Hypotension was treated by ephedrine 5 mg and bradycardia by atropine 0.5 mg intravenously. Desaturation or apnea was treated with supplemental oxygen (10 L/min) via a face mask and respiratory assistance was provided by a chin lift or jaw thrust maneuver or assisted mask ventilation. Laryngeal mask airway insertion or tracheal intubation was allowed in the event of persistent apnea according to the decision of the attending anesthesiologists. 

The Ramsay Sedation Scale (RSS) could not be used because the patients in the VR group were watching the VR program [15]. The incidence of nausea (≥3 on an 11-point numeric rating scale reported by the patient where 0 is no nausea and 10 is the worst nausea imaginable) and vomiting during surgery and in the recovery room was documented. During their stay in the recovery room, the patients were presented with score table again and asked to rate their satisfaction when they became completely alert. If the patient was confused by the residual effect of midazolam, the scoring of satisfaction was performed after transfer to the general ward. The patients were also asked about their memory of the surgical procedure or procedural pain they experienced during surgery (measured a 0–10 numeric rating scale where 0 is no pain and 10 is the worst pain imaginable). Length of stay in the recovery room was recorded. The criteria for discharge to the recovery room in our hospital is a modified Aldrete score ≥9 (Supplemental Table S1) [16]. 

Finally, considering all the variables mentioned above, the incidence of a composite of optimal patient, anesthesia and surgical conditions was determined for each study participant when all the following five criteria were satisfied: (1) the patient did not complain of discomfort, did not move unnecessarily, did not try to detach the monitoring devices, and remained lying still; (2) desaturation or apnea did not occur; (3) the patient did not need assisted ventilation, laryngeal mask airway insertion, or tracheal intubation; (4) there was no need for rescue medication; and (5) hypotension or bradycardia did not occur or occurred only once. 

### 2.6. Statistical Analysis

No previous study has compared satisfaction scores between VR and drug sedation groups. We calculated that a sample size of 17 subjects would be needed in each group based on a power analysis assuming a mean difference in the satisfaction score of 1.0 and a standard deviation of 1.0. Allowing for a dropout rate of 10%, 37 patients were required. 

The final results were evaluated by intention-to-treat analysis. Categorical variables were analyzed by the chi-squared or Fisher’s exact test. Continuous variables were compared using the unpaired *t* test or Mann-Whitney *U* test according to the normality of the variables, and mean differences were calculated with the confidence intervals (CIs). The Shapiro-Wilk test and Q-Q plots were used to test the normality of the data distribution. The Cohen’s kappa statistic was used to determine the agreement beyond chance between the two surgeon’s and two anesthesiologist’s satisfaction scores. The agreement was interpreted according to the following scale: 0.80 to 1.00, very good; 0.60 to 0.80, good; 0.40 to 0.60, moderate; 0.20 to 0.40, fair; less than 0.20, poor agreement. *p* < 0.05 was considered statistically significant. All statistical analyses were performed using SPSS Version 22.0 (IBM Corp., Armonk, NY, USA). 

## 3. Results

**Figure 1:**
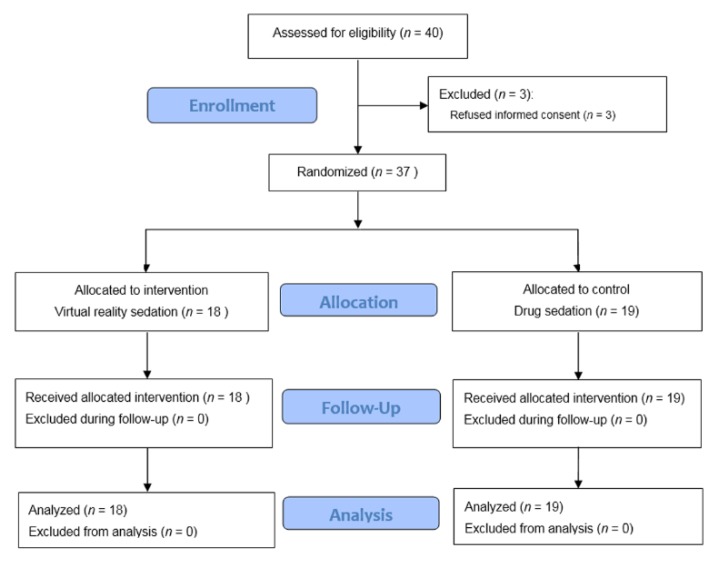
Flow diagram of the study according to CONSORT 2010.

Of the 40 patients assessed for eligibility to be included in this study (Figure 1), three patients refused to participate. The remaining 37 patients were enrolled and randomized to the VR group (*n* = 18) or to the sedation group (*n* = 19). All 37 patients finished the study protocol and were included in the analysis. No patient in the VR group asked to stop watching the VR program and no rescue medication was administered in either study group. All patients were able to report their satisfaction score in the recovery room. There were no missing data for any of our study variables. The patient characteristics at baseline are shown in Table 2. The patient demographics and comorbidity were comparable. The duration of surgery and weight of the prostate were not different between the groups.

**Figure 2:**
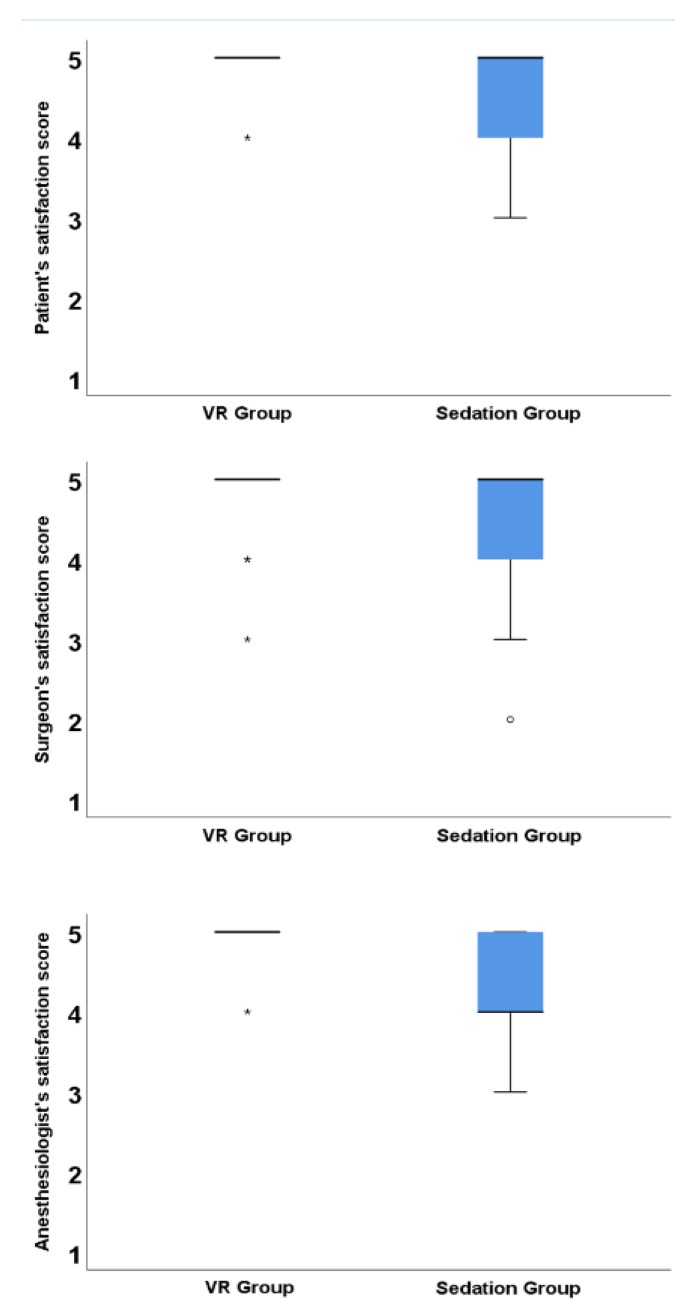
Box-and-whisker plots of the satisfaction scores of patients, surgeons, and anesthesiologists. Thick horizontal bars, boxes, and error bars represent the median, 25th, 75th, 10th and 90th percentile. VR group = Virtual Reality group, 1 = extremely dissatisfied, 2 = dissatisfied, 3 = undecided, 4 = satisfied, 5 = extremely satisfied, ⸰ means outlier and * means extreme outlier. Extreme outlier is determined when outliers is located distal to 1.5× interquartile range from 25 or 75 percentile.

The satisfaction scores failed the normality test. The distribution of the satisfaction scores of the patients and anesthesiologists were significantly different between the groups (*p* = 0.025 and *p* = 0.001, respectively), while the score of the surgeons was not significantly different (Figure 2). The incidence of extreme satisfaction (satisfaction score 5) for patients and anesthesiologists was significantly higher in the VR group than in the sedation group (patients, *n* = 17, 94.4% in the VR group vs. *n* = 12, 63.2% in the sedation group, *p* = 0.042). Two independent assessors of the satisfaction score showed very good agreement beyond that expected by chance (surgeons, kappa = 0.874, 95% CI = 0.730–1.000, *p* < 0.001; anesthesiologists, kappa = 0.944, 95% CI = 0.739–1.000, *p* < 0.001). There were two cases of discrepancy in reporting the satisfaction score of the surgeons and one case of discrepancy for the score of the anesthesiologists. The difference was one-point and a higher score was selected for all cases. 

Table 3 shows the comparison of outcome variables between the groups. Most patients did not move inadvertently during surgery in the VR group. There were significantly more patients who developed apnea in the sedation group than in the VR group (*n* = 1, 5.6% in group VR vs. *n* = 7, 36.8% in the sedation group, *p* = 0.042, risk difference (95% CI) −0.34, (−0.09 to −0.58)). One patient in the sedation group received assisted mask ventilation. Two patients in the VR group fell asleep while watching the VR program. They did not receive propofol and their group assignment was not changed. The hemodynamic variables during surgery, including the incidence of hypotension and bradycardia, were not different between the groups. The duration of stay in the recovery room was not different between the groups. 

The incidence of optimal patient, anesthesia and surgical conditions was significantly higher in the VR group than in the sedation group (*n* = 17, 94.4% in VR group vs. *n* = 13, 68.4% in the sedation group, *p* = 0.043, risk difference (95% CI) 0.17 (−0.08 to 0.42)). There was no harmful or unintended effect of the study interventions other than the reported outcomes. 

## 4. Discussion

We conducted a randomized controlled trial comparing VR distraction and drug sedation in patients undergoing endoscopic prostatectomy under spinal anesthesia. The satisfaction score of the anesthesiologists was significantly higher in the VR group than that in the sedation group. The incidences of extreme satisfaction reported by the patients and anesthesiologists were significantly higher in the VR group than those in the sedation group. Our study results suggest that VR distraction may be preferable for both patients and anesthesiologists during endoscopic urologic surgery under spinal anesthesia. 

VR has been studied for its potential use in simulated medical training [17,18], pain control for medical procedures and physical trauma [19,20,21], and rehabilitation of patients with stroke or Parkinson’s disease [22,23]. Recently, VR distraction has been studied in regional anesthesia to assess its sedation and analgesic-sparing effects [12,24]. However, the studies included only a small number of patients as a pilot and feasibility study or were retrospective in nature. Furthermore, the commercial VR programs used did not aim to improve patient relaxation and included no verbal narration inducing relaxation. In this prospective randomized controlled trial, we used a commercially available VR sedation program specifically designed for perioperative settings to evaluate participants’ satisfaction. The supplemental text and figures present a glimpse of the program, including the narration and a silent scene of the three-dimensional undersea environment inducing relaxation and sedation. Our study may have value as the first trial to investigate the effect of a specific VR program as a single modality to replace drug sedation during spinal anesthesia. 

The incidence of extreme satisfaction reported by both patients and anesthesiologists was higher in the VR group than in the sedation group and the anesthesiologist’s satisfaction scores were significantly higher in the VR group. However, there was no difference in the surgeon’s satisfaction scores between the groups. From a surgeon’s perspective, the respiratory depression caused by midazolam may not influence the surgical conditions, although the patient may move purposelessly during respiratory depression or snoring. The incidence of apnea caused by respiratory depression also influenced the rate of adequate sedation in our study. 

The persistent penile erection that occurs during HOLLEP may interfere with the surgical procedure because any movement of the cystoscope could be interrupted. Drug sedation with midazolam may cause sleep-related penile erection [25], whereas VR distraction may avoid this sleep-related problem and contribute to better surgical conditions. A previous study that monitored penile tumescence reported that sleep-related erectile episodes occurred in nearly all patients who received midazolam. Therefore, we included penile erection in our criteria for the surgeon’s satisfaction score (Supplemental Table S1). However, there was no difference in this regard between the groups, suggesting that our study was not adequately powered to detect the small difference in the incidence of penile erection during midazolam sedation under spinal anesthesia. 

We compared VR distraction with drug sedation with midazolam. However, this benzodiazepine has a relatively long duration of action and is not a preferred sedative nowadays compared with propofol or dexmedetomidine. Our study results indeed showed a high incidence of apnea during sedation with midazolam. Propofol or dexmedetomidine are also used frequently for procedural sedation [26,27,28,29]. Propofol is associated with rapid recovery, although arterial hypotension may develop in response to suppression of reflex tachycardia. Sedation with dexmedetomidine provides improved analgesia and causes less respiratory depression than other sedatives, including benzodiazepines and propofol [30,31]. Further studies are required to compare VR sedation with drug sedation using propofol or dexmedetomidine. 

Our study has several limitations. First, the primary outcome variable was the patient-reported satisfaction score. Our sample size was calculated based on the difference in this score. Other secondary variables were not considered when calculating the sample size; therefore, our study may have lacked statistical power for the secondary outcomes. Furthermore, the satisfaction score of the patient was reported by different patients who were not blind to the group assignment. To decrease the patient-dependent reporting bias, our patients determined their satisfaction score according to a prespecified scoring scale (Table 1). However, our score was not validated in any previous study. Although the satisfaction scores of the surgeons and anesthesiologists were reported by two personnel after discussion and their kappa statistics were analyzed, the patient scores could not be analyzed in that manner. Second, our study was a small single-center trial, so its external validity may be limited. The effect of VR distraction may be different in different surgical settings and age groups. Third, the attending anesthesiologists and patients could not be blinded to group assignment, which could have confounded the satisfaction scores of anesthesiologists and patients.

## 5. Conclusions 

Our study results suggest that VR distraction is preferable to pharmacologic sedation with midazolam in terms of patient’s and anesthesiologist’s satisfaction and avoiding the respiratory side effects of midazolam during endoscopic urologic surgery under spinal anesthesia in patients with ASA physical status classification of I–III. Further studies are required to detect a true clinical advantage of VR distraction over drug sedation. 

## Figures and Tables

**Table 1:** Satisfaction score.

Rater	Score	Description
Surgeon	1 (extremely dissatisfied)	Any of the following: 1. Patients involuntarily moved or repeatedly spoke, which made the surgical procedure stop more than twice. 2. Persistent penile erection (3 or more episode lasting >10 s) that significantly interfered with the surgical procedure and required phenylephrine injection.
	2 (dissatisfied)	Any of the following: 1. Patients involuntarily moved or repeatedly spoke, which made the surgical procedure stop once. 2. Persistent penile erection (2 or more episodes >10 s) that interfered with the surgical procedure, but not used phenylephrine.
	3 (undecided)	Any of the following: 1. Patients involuntarily moved or spoke a little, but no interference with the surgical procedure. 2. Transient penile erection (<10 s) that did not interfere with surgical procedure significantly.
	4 (satisfied)	All of the following: 1. No patient’s voluntary movement or speaking. No interference with the surgical procedure. 2. Transient penile erection (<10 s) which did not interfere with the surgical procedure at all.
	5 (extremely satisfied)	All of the following: 1. No patient’s voluntary movement or speaking. No interference with the surgical procedure. 2. No penile erection at all.
Patient	1 (extremely dissatisfied)	Any of the following: 1. Patients recalled all steps of the surgical procedure. 2. Patients felt severe pain during surgery (NRS ≥ 7). 3. Patients repeatedly moved due to discomfort.
	2 (dissatisfied)	Any of the following: 1. Patients recalled about two-thirds of the surgical procedure time (including recall of inserting and withdrawing the fiberoptic scope into the urethra). 2. Patients felt moderate pain (NRS of 4 to 6). 3. Patients moved several times due to discomfort.
	3 (undecided)	Any of the following: 1. Patients recalled less than one-third of the surgical procedure time. 2. Patients felt mild pain (NRS of 2 to 3).
	4 (satisfied)	All of the following: 1. No patient’s recall of surgery. 2. No pain at all. 3. Patients felt comfortable during surgery.
	5 (extremely satisfied)	All of the following: 1. No patient’s recall of surgery. 2. No pain at all. 3. Patients felt extreme comfort and were not concerned about the lithotomy position and surgery at all.
Anesthesiologist	1 (extremely dissatisfied)	Any of the following: 1. Patients involuntarily moved 3 or more times and repeatedly spoke which interfered the surgical procedure. 2. More than three events of apnea. 3. Desaturation (SpO_2_ < 90% more than 5 s) more than twice. 4. Assisted ventilation required due to desaturation and apnea.
	2 (dissatisfied)	Any of the following: 1. Patients involuntarily moved or spoke 1 to 2 times, which interfered the surgical procedure. 2. Apnea developed twice. 3. Desaturation (SpO_2_ < 90% more than 5 s) once. 4. Oral or nasal airway insertion due to desaturation or apnea.
	3 (undecided)	All of the following: 1. Patients involuntarily moved or spoke 1 to 2 times, but did not interfere with the surgery. 2. Apnea developed once but did not require oral or nasal airway insertion or assisted ventilation.
	4 (satisfied)	All of the following: 1. No patient’s voluntary movement or speaking. 2. Apnea developed once but did not require oral or nasal airway insertion or assisted ventilation.
	5 (extremely satisfied)	All of the following: 1. No patient’s voluntary movement or speaking. 2. No Apnea.

NRS = Numerical Rating Scale.

**Table 2:** Baseline characteristics of patients and surgical characteristics.

Variables	VR Group	Sedation Group
Case number, *n*	18	19
Age, years	69 (65–70)	69 (63–72)
Weight, kg	69 (64–73)	64 (62–69)
Height, cm	167 (163–170)	167 (162–170)
Body-mass index, kg m^−2^	24.8 (23.3–26.9)	23.9 (21.3–25.1)
ASA PS, 1/2/3	7/7/4	9/7/3
Underlying disease (*n*)		
Hypertension	8 (44.4)	8 (42.1)
Diabetes Mellitus	3 (16.7)	3 (15.8)
Angina pectoris	3 (15.8)	3 (16.7)
Stroke	2 (11.1)	-
Chronic obstructive pulmonary disease	-	1 (5.3)
Chronic kidney disease	2 (11.1)	-
Prostate weight (g)	65 (38–98)	75 (39–110)
Duration of anesthesia (min)	65 (55–100)	65 (60–85)
Duration of surgery (min)	40 (35–75)	45 (30–60)
Fluid administration (mL)	200 (60–200)	100 (70–150)
Colloid administration (mL)	-	-

The values are expressed as the median (interquartile range) or number (%). VR Group = virtual reality group; ASA PS = American society of anesthesiologist physical status classification. *p*-values are the results of Mann-Whitney U test for continuous variables and Fisher exact test for categorized variables.

**Table 3:** Sedation-related characteristics.

Variables	VR Group	Sedation Group	*p*-Value
Case number, *n*	18	19	
Intraoperative variables			
Patients who do not move involuntarily during surgery, *n*	16 (88.9)	13 (68.4)	0.001
Patients who requested to stop watching VR, *n*	0	-	
Midazolam administration, mg	4 (4–6)	-	
Administration of rescue sedative, *n*	0	0	
Desaturation (SpO_2_ < 90%, more than 5 s), *n*	0	1 (5.3)	0.999
Apnea (flat ETco_2_, more than 5 s)			
Develop, *n*	1 (5.6)	7 (36.8)	0.042
Frequency in the patients with apnea, range	1–2	1–5	
Assisted mask ventilation, *n*	0	1	0.999
Conversion to general anesthesia, *n*	0	0	
Ephedrine administration			
Incidence, *n*	2 (11.1)	6 (31.6)	0.232
Dose, median (IQR) (range), mg	0 [0,0] (5–10)	0 (0–5) (5–10)	0.313
Atropine administration			
Incidence, *n*	1 (5.6)	4 (21.1)	0.340
Dose, median (IQR) (range), mg	0 [0,0] (0–0.5)	0 (0–0) (0–0.5)	0.425
Nausea (≥3 of numerical rating scale), *n*	0	2	0.486
Vomiting. *n*	0	0	
Satisfaction score as a continuous variable			
Patient	5 (5–5)	5 (4–5)	0.105
Surgeon	5 (5–5)	5 (4–5)	0.558
Anesthesiologist	5 (5–5)	4 (4–5)	0.005
Incidence of extreme satisfaction			
Patient	17 (94.4)	12 (63.2)	0.042
Surgeon	14 (77.8)	13 (68.4)	0.714
Anesthesiologist	17 (94.4)	8 (42.1)	0.001
Recovery room parameters			
Remember the operative procedure, *n*	0	3 (15.8)	0.230
Felt procedural pain during surgery, *n*	0	0	
Duration of recovery room stay, min	27 (21–44)	29 (22–53)	0.620
Nausea (≥3 of numerical rating scale), *n*	1	2	0.999
Vomiting, *n*	0	0	
Optimal patient, anesthesia, and surgical condition, *n*	17 (94.4)	12 (63.2)	0.042

The values are expressed as the median (interquartile range) or number (%). VR Group = Virtual reality group; ASA PS = American society of anesthesiologist physical status classification, ETco_2_ = end-tidal carbon dioxide. *P*-values are the results of Mann-Whitney U test for continuous variables and Fisher exact test for categorized variables. Hypotension was determined when heart rate <50/min or 30% or more decrease from baseline. Bradycardia was determined when heart rate <50/min or 30% or more decrease from baseline.

## Supplementary Materials

The following are available online at http://www.mdpi.com/2077-0383/8/1/2/s1, Supplemental Table S1: Modified Aldrete Score, Supplemental Figure S1: Patient during Holmium laser enucleation of the prostate under spinal anesthesia with virtual reality distraction, Supplemental Figure S2: A Screenshot of ‘Aqua 30’, Supplemental Figure S3: A Screenshot of ‘Aqua 30’, Supplemental Figure S4: A Screenshot of ‘Aqua 30’, Supplemental Text S1: Script of ‘Aqua 30’ (Introduction part only).

## References

[1] Li A., Montano Z., Chen V.J., Gold J.I. (2011). Virtual reality and pain management: Current trends and future directions. Pain Manag..

[2] Zeng N., Pope Z., Lee J.E., Gao Z. (2018). Virtual reality exercise for anxiety and depression: A preliminary review of current research in an emerging field. J. Clin. Med..

[3] Jensen J.K., Dyre L., Jorgensen M.E., Andreasen L.A., Tolsgaard M.G. (2018). Simulation-based point-of-care ultrasound training: A matter of competency rather than volume. Acta Anaesthesiol. Scand..

[4] Zaveri P.P., Davis A.B., O’Connell K.J., Willner E., Aronson Schinasi D.A., Ottolini M. (2016). Virtual reality for pediatric sedation: A randomized controlled trial using simulation. Cureus.

[5] Jensen K., Ringsted C., Hansen H.J., Petersen R.H., Konge L. (2014). Simulation-based training for thoracoscopic lobectomy: A randomized controlled trial: Virtual-reality versus black-box simulation. Surg. Endosc..

[6] Kulcsar Z., O’Mahony E., Lovquist E., Aboulafia A., Sabova D., Ghori K., Iohom G., Shorten G. (2013). Preliminary evaluation of a virtual reality-based simulator for learning spinal anesthesia. J. Clin. Anesth..

[7] Latif R.K., Bautista A., Duan X., Neamtu A., Wu D., Wadhwa A., Akça O. (2016). Teaching basic fiberoptic intubation skills in a simulator: Initial learning and skills decay. J. Anesth..

[8] Gold J.I., Kim S.H., Kant A.J., Joseph M.H., Rizzo A.S. (2006). Effectiveness of virtual reality for pediatric pain distraction during IV placement. Cyberpsychol. Behav..

[9] Furman E., Jasinevicius T.R., Bissada N.F., Victoroff K.Z., Skillicorn R., Buchner M. (2009). Virtual reality distraction for pain control during periodontal scaling and root planing procedures. J. Am. Dent. Assoc..

[10] Morris L.D., Louw Q.A., Grimmer-Somers K. (2009). The effectiveness of virtual reality on reducing pain and anxiety in burn injury patients: A systematic review. Clin. J. Pain.

[11] Ryu J.H., Park J.W., Nahm F.S., Jeon Y.T., Oh A.Y., Lee H.J., Kim J.H., Han S.H. (2018). The effect of gamification through a virtual reality on preoperative anxiety in pediatric patients undergoing general anesthesia: A prospective, randomized, and controlled trial. J. Clin. Med..

[12] Chan P.Y., Scharf S. (2017). Virtual reality as an adjunctive nonpharmacological sedative during orthopedic surgery under regional anesthesia: A pilot and feasibility study. Anesth. Analg..

[13] Angelini G., Ketzler J.T., Coursin D.B. (2001). Use of propofol and other nonbenzodiazepine sedatives in the intensive care unit. Crit. Care Clin..

[14] Shafer A. (1998). Complications of sedation with midazolam in the intensive care unit and a comparison with other sedative regimens. Crit. Care Med..

[15] Ramsay M.A., Savege T.M., Simpson B.R., Goodwin R. (1974). Controlled sedation with alphaxalone-alphadolone. Br. Med. J..

[16] Aldrete J.A. (1998). Modifications to the postanesthesia score for use in ambulatory surgery. J. PeriAnesth. Nurs..

[17] Nagendran M., Gurusamy K.S., Aggarwal R., Loizidou M., Davidson B.R. (2013). Virtual reality training for surgical trainees in laparoscopic surgery. Cochrane Database Syst. Rev..

[18] Piromchai P., Avery A., Laopaiboon M., Kennedy G., O’Leary S. (2015). Virtual reality training for improving the skills needed for performing surgery of the ear, nose or throat. Cochrane Database Syst. Rev..

[19] Teeley A.M., Soltani M., Wiechman S.A., Jensen M.P., Sharar S.R., Patterson D.R. (2012). Virtual reality hypnosis pain control in the treatment of multiple fractures: A case series. Am. J. Clin. Hypn..

[20] Patterson D.R., Jensen M.P., Wiechman S.A., Sharar S.R. (2010). Virtual reality hypnosis for pain associated with recovery from physical trauma. Int. J. Clin. Exp. Hypn..

[21] Enea V., Dafinoiu I., Opris D., David D. (2014). Effects of hypnotic analgesia and virtual reality on the reduction of experimental pain among high and low hypnotizables. Int. J. Clin. Exp. Hypn..

[22] Dockx K., Bekkers E.M., Van den Bergh V., Ginis P., Rochester L., Hausdorff J.M., Mirelman A., Nieuwboer A. (2016). Virtual reality for rehabilitation in Parkinson’s disease. Cochrane Database Syst. Rev..

[23] Laver K.E., Lange B., George S., Deutsch J.E., Saposnik G., Crotty M. (2017). Virtual reality for stroke rehabilitation. Cochrane Database Syst. Rev..

[24] Pandya P.G., Kim T.E., Howard S.K., Stary E., Leng J.C., Hunter O.O., Mariano E.R. (2017). Virtual reality distraction decreases routine intravenous sedation and procedure-related pain during preoperative adductor canal catheter insertion: A retrospective study. Korean J. Anesthesiol..

[25] Song Y.S., Song E.S., Lee K.H., Park Y.H., Shin W.C., Ku J.H. (2006). Sleep-related nocturnal erections and erections during midazolam-induced sedation in healthy young men. Int. J. Impot. Res..

[26] Sriganesh K., Reddy M., Jena S., Mittal M., Rao G.S.U. (2015). A comparative study of dexmedetomidine and propofol as sole sedative agents for patients with aneurysmal subarachnoid hemorrhage undergoing diagnostic cerebral angiography. J. Anesth..

[27] Ryu J.H., Park S.J., Park J.W., Kim J.W., Yoo H.J., Kim T.W., Hong J.S., Han S.H. (2017). Randomized clinical trial of immersive virtual reality tour of the operating theatre in children before anaesthesia. Br. J. Surg..

[28] Muller S., Borowics S.M., Fortis E.A., Stefani L.C., Soares G., Maguilnik I., Breyer H.P., Hidalgo M.P., Caumo W. (2008). Clinical efficacy of dexmedetomidine alone is less than propofol for conscious sedation during ERCP. Gastrointest. Endosc..

[29] Kim K.H. (2014). Safe sedation and hypnosis using dexmedetomidine for minimally invasive spine surgery in a prone position. Korean J. Pain.

[30] Arain S.R., Ebert T.J. (2002). The efficacy, side effects, and recovery characteristics of dexmedetomidine versus propofol when used for intraoperative sedation. Anesth. Analg..

[31] Ter Bruggen F., Eralp I., Jansen C.K., Stronks D.L., Huygen F. (2017). Efficacy of dexmedetomidine as a sole sedative agent in small diagnostic and therapeutic procedures: A systematic review. Pain Pract..

