# Supplementary material for: Virtual Reality Distraction during Endoscopic Urologic Surgery under Spinal Anesthesia: A Randomized Controlled Trial
Source: J Clin Med. 2018 Dec 20;8(1):2. doi: 10.3390/jcm8010002 (PMC6352098; doi:10.3390/jcm8010002)
Supplement: Supplementary file 1 [file jcm-08-00002-s001.pdf]

Virtual reality distraction during endoscopic urological surgery under spinal anesthesia: A randomized controlled trial

Jee Youn Moon, Jungho Shin , Jaeyeon Chung, Sang-Hwan Ji, Soohan Ro, Won Ho Kim

Supplemental Materials

Supplemental Figure S1. Patient during Holmium laser enucleation of the prostate under spinal anesthesia with virtual reality distraction.

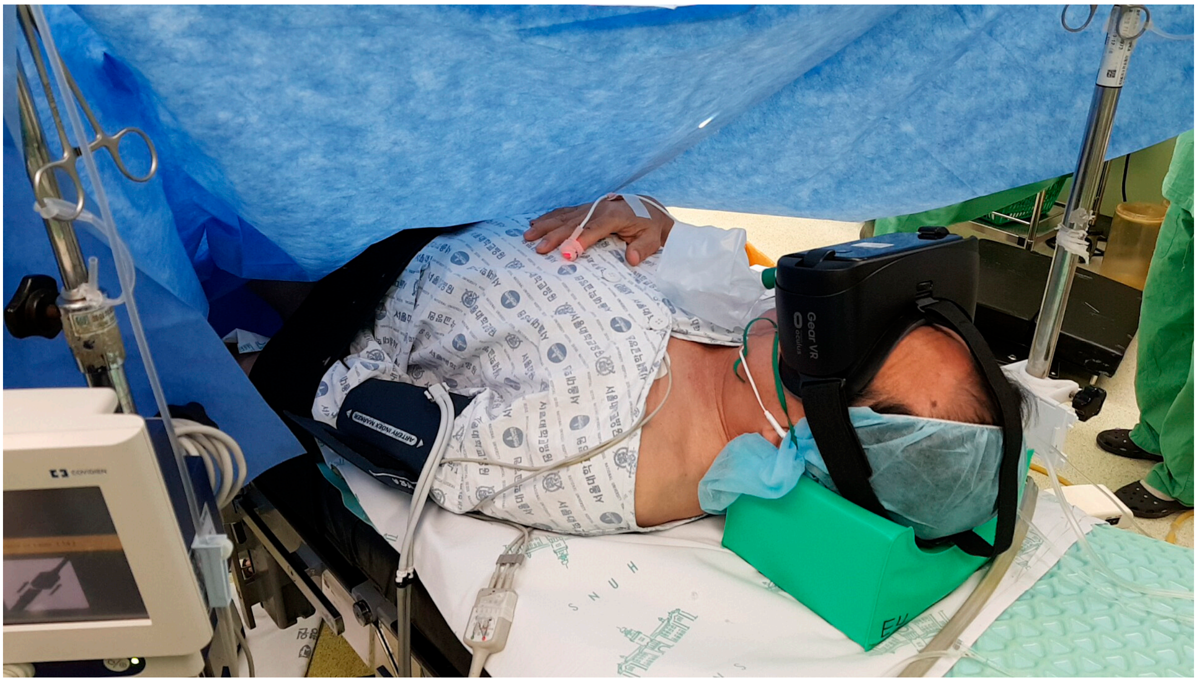

Supplemental Figure S2. A Screen shot of 'Aqua 30'

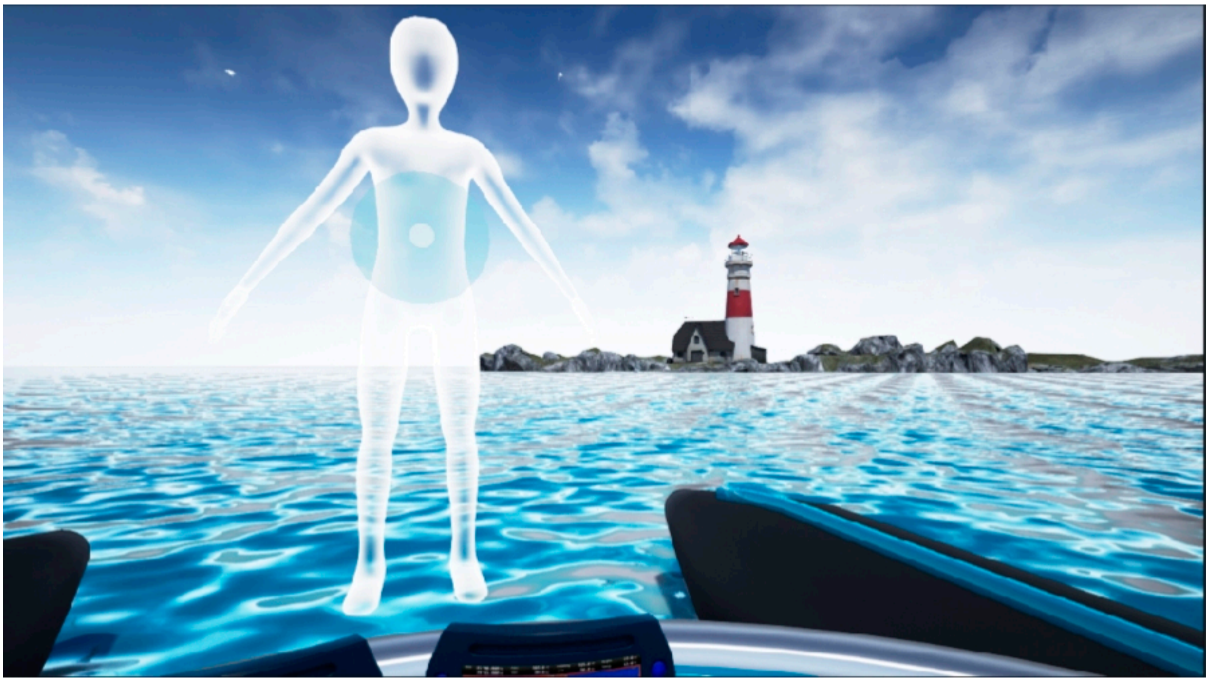

Supplemental Figure S3. A Screen shot of ‘Aqua 30’

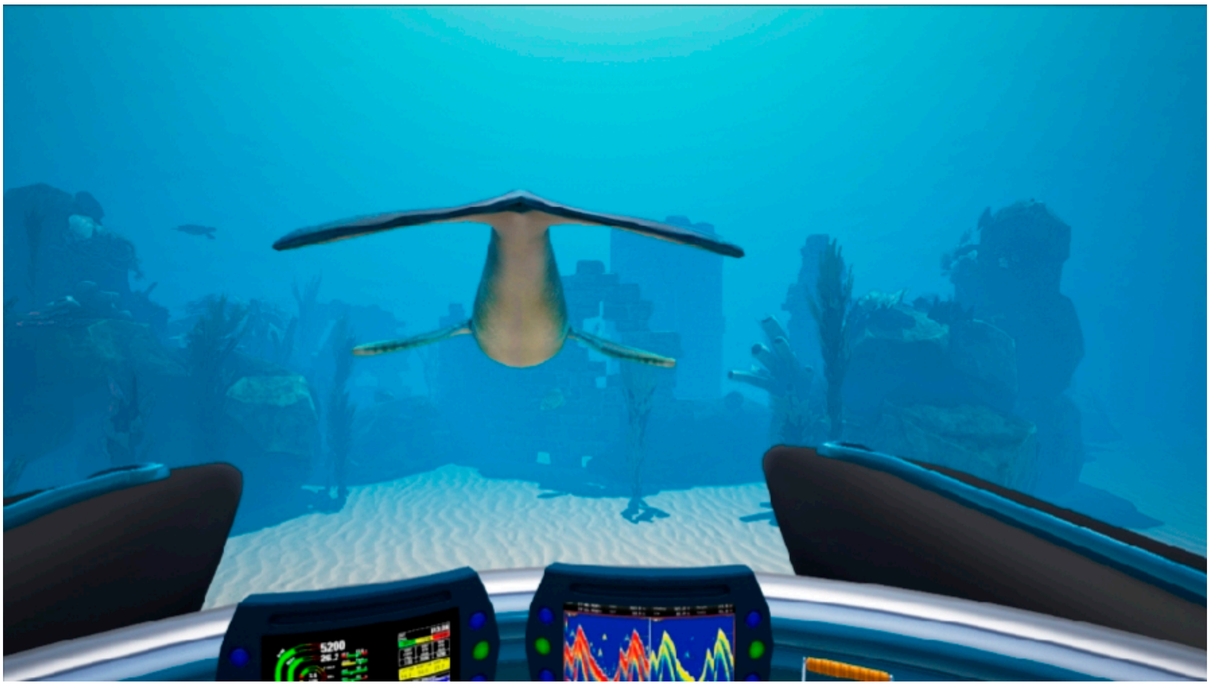

Supplemental Figure S4. A Screen shot of ‘Aqua 30’

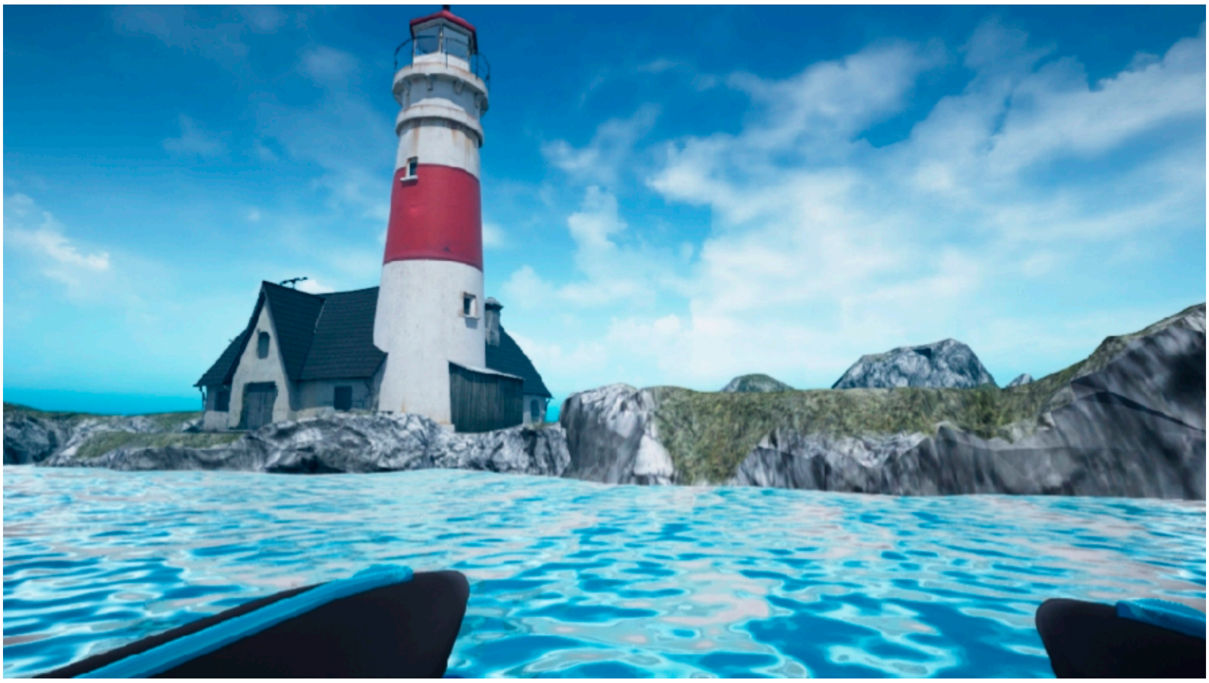

Supplemental Text S1. Script of ‘Aqua 30’ (Introduction part only)

Welcome to Aqua, your relaxing underwater experience!

This experience has been created for you. Take the time to look around you. You will be able to discover the whole virtual environment. Go ahead and have some fun, be curious, and play around as you experience all the beneficial effects of Aqua.

You should see a dashboard right in front of you and a lighthouse on your right. If this is not the case, or if the volume of the audio is too loud or soft, let us know now and we will adapt it for you.

During this experience, you will discover, little by little, how you can use your intuition, and discover resources inside of you which will help you create more comfort. Get in touch with peace and tranquility. You will be able to use this tranquility whenever you wish, or whenever you feel the need for it in the future.

During the first part of the experience, be just a spectator. Explore and discover the environment and calming experience around you. You do not need to do anything in particular. Just let your all attention on this experience. Deeply focused. Be open to this experience. Curious like a child playing a game for the first time.

……(omitted)

Supplemental Table S1. Modified Aldrete Score.

Activity: able to move voluntarily or on command	Score
4 extremities	2
2 extremities	1
0 extermities	0
Respiration	
Able to deep breathe and cough freely	2
Dyspnea, shallow or limited breathing	1
Apneic	0
Circulation	
BP < 20 % of preanesthesia level	2
BP between 20 and 50% of preanesthesia level	1
BP > 50% of preanesthesia level	0
Consciousness	
Fully awake	2
Arousable on calling	1
Not responding	0
O_2 saturation	
Able to maintain O_2 saturation > 90% on room air	2
Needs O_2 inhalation to maintain O_2 saturation > 90%	1
O_2 saturation <90% even when O_2 supplementation	0
